# Diagnosis of Niemann-Pick disease type C with 7-ketocholesterol screening followed by NPC1/NPC2 gene mutation confirmation in Chinese patients

**DOI:** 10.1186/1750-1172-9-82

**Published:** 2014-06-10

**Authors:** Huiwen Zhang, Yu Wang, Na Lin, Rui Yang, Wenjuan Qiu, Lianshu Han, Jun Ye, Xuefan Gu

**Affiliations:** 1 Department of Pediatric Endocrinology and Genetic Metabolism, Xinhua Hospital, Shanghai Institute for Pediatric Research, Shanghai Jiao Tong University School of Medicine, Shanghai, China

**Keywords:** Niemann-Pick type C, 7-ketocholesterol, *NPC1* gene, *NPC2* gene

## Abstract

**Background:**

It has been reported that oxidation product of cholesterol, 7-ketocholesterol, increases in plasma of patients with NP-C. Previously, we established a rapid test to determine the plasma 7-ketocholesterol level and found it elevated significantly in patients with acid sphingomyelinase deficient NPD and NP-C disease.

**Methods:**

Individuals randomly referred to our outpatient clinics in the past two years for hepatosplenomegaly or isolated splenomegaly, who have been excluded as acid sphingomyelinase deficient NPD or Gaucher disease, and individuals with newborn cholestasis, psychomotor regression/retardation, were screened for plasma 7-ketocholesterol level. Individuals with high 7-ketocholesterol level were then analyzed for *NPC1* and *NPC2* gene mutation to confirm the accuracy of NP-C diagnosis.

**Results:**

By screening the plasma 7-ketocholesterol of suspect individuals, 12 out of 302 (4%) had shown remarkable high levels compared with reference. All these twelve individuals were subsequently confirmed to be NP-C by DNA analysis of *NPC1* and *NPC2* genes, with the early infantile form (n = 7), the late infantile form (n = 1), the juvenile form (n = 1) and the adult form (n = 1). Furthermore, two NP-C patients without observable neuropsychiatric disability were picked up through this procedure. Only one patient had NP-C due to *NPC2* gene mutations, with the rest due to *NPC1* gene mutations. We found that in NP-C patients AST was usually mildly elevated and ALT was in a normal range when jaundice was not present. In total, 22 mutant alleles were identified in the *NPC1* gene, including six novel small deletions/insertions, e.g., c.416_417insC, c.1030delT, c.1800delC, c.2230_2231delGT, c.2302_2303insG, and c.2795dupA; seven novel exonic point mutations, c.1502A>T (p.D501V), c.1553G>A (p.R518Q), c.1832A>G (p.D611G), c.2054T>C (p.I685T), c.2128C>T(p.Q710X), c.2177G>C (p.R726T), c.2366G>A (p.R789H), and one novel intronic mutation c.2912-3C>G. Small deletions/insertions constituted nearly half of the mutant alleles (10/22, 45%), indicating a unique mutation spectrum in this cohort of Chinese NP-C patients.

**Conclusion:**

Our data confirm in a clinical setting that screening plasma 7-ketocholesterol is an efficient and practical diagnostic tool to identify NP-C patients from suspect individuals. Patients without neuropsychological involvement could also be identified by this method therefore allowing an opportunity for earlier treatment.

## Introduction

Niemann-Pick disease type C (NP-C, OMIM 257220, 607625) is a rare autosomal recessive disease belonging to the family of lysosomal storage disorders. Mutations in one of two lysosome-related genes have been identified as accounting for the occurrence of this disease. One is a large lysosomal integral membrane protein NPC1 [1], which transfers low density lipoprotein-derived free cholesterol out of lysosomes or late endosomes [2]. The other is a small lysosomal soluble protein NPC2 [3], which functions in concert with NPC1 [4]. Defects of these two proteins result in sequestration of mainly unesterified cholesterol, as well as other sphingolipids in the late endosomes/lysosomes at the cellular level, ultimately leading to a clinical neurovisceral manifestation characterized by hepatosplenomegaly and severe progressive neurological dysfunction, which includes vertical supranuclear gaze palsy, ataxia, dystonia, dysarthria, dysphagia, gelastic cataplexy, and epileptic seizures. According to age at onset of neurological manifestations, NP-C patients are categorized into five forms, i.e., perinatal, early-infantile, late-infantile, juvenile, and adult forms [5].

Clinical manifestations of NP-C are usually not disease-specific and are extremely variable. Taking the perinatal form as an example, newborns with this form of NP-C typically have cholestasis and hepatosplenomegaly. At a North American medical centre, in all infants evaluated for neonatal cholestasis, NP-C was identified in 8% of them, with the rest being affected by other disorders [6]. In a multicentre genetic screening study evaluating the frequency of NP-C among patients who shared common neurological and psychiatric symptoms, only three of 256 adults (1.2%) were confirmed to be NP-C [7]. In the literature NP-C has been misdiagnosed as Wilson disease [8] and Gaucher disease [9]. Generally, there was a long lag time between the initial onset of neurological manifestations and definite diagnosis [10]. Miglustat has been shown to be an effective therapy for NP-C [11] and a new drug, 2-hydroxypropyl-beta-cyclodextrin (Cyclodextrin), is being tested in a clinical trial for this disease. Earlier treatment with Miglustat has been shown to be associated with a better prognosis [12]. Thus, prompt and accurate diagnosis is becoming increasingly important.

NP-C is pan ethnic and is estimated with an incidence of 1:120,000 live births [2,13]. Numerous patients have been reported in Western countries [10,14-16], but only approximately 10 Chinese patients have been confirmed to have this disorder [17,18]. It is highly likely that a large portion of Chinese NP-C patients are undiagnosed, which is possibly due to both the extremely variable presentation of NP-C and the complex procedure used to biochemically demonstrate abnormal intracellular cholesterol homeostasis in cultured fibroblasts.

It was reported that cholesterol oxidation products, 7-ketocholesterol (7-KC) and cholestan-3β,5α,6β-triol (3β,5α,6β-triol), were markedly increased in the plasma of human NPC1 subjects; therefore, these products could be used as non-invasive and highly sensitive biomarkers for NP-C disease [19,20]. But the diagnostic value of these biomarkers has not been evaluated in the clinical settings. Previously, we established a method for rapid plasma 7-KC detection [21], and found that high 7-KC levels could not differentiate between acid sphingomyelinase (ASM) deficiency and NP-C. Clinically, ASM deficiency belongs to the differential diagnosis of NP-C, at least at a certain stage of the disease. High 7- KC levels in patients with systemic symptoms indicate the patients should be further analyzed for ASM activity test, followed by *SMPD1* gene mutation test in case of deficient ASM activity, or *NPC1*/*NPC2* gene mutation test in case of normal ASM activity .

We have set up an ASM activity test to diagnose ASM deficiency in our lab among individuals with hepatospleomagaly or isolated splenomegaly [22]. We speculated that there may be NP-C patients in those without a clear diagnosis after ASM and glucocerebrosidase assay. So we use the biomarker 7-KC to screen these patients, as well as newborns with cholestasis, and individuals referred to our centre for psychomotor regression/retardation. Individuals with elevated 7-KC were followed by molecular genetic testing of *NPC1* and *NPC2* genes to confirm the diagnosis. Here we report the clinical and molecular characteristics of 12 new Chinese NP-C patients identified through this procedure in the past two years.

## Materials and methods

### Compliance with ethics guidelines

All procedures were in accordance with the ethical standards of committee on human experimentation in Shanghai Xinhua hospital (approval number XHEC-D-2011-017) and with the Helsinki Declaration of 1975, as revised in 2012. Informed consent was obtained from each patient for inclusion in the study.

### Indications to be included in 7-KC screening

Individuals randomly referred to our department for newborn cholestasis (group A), hepatosplenomegaly/hepatomegaly/splenomegaly (group B), psychomotor regression/retardation (group C), in the past two years were recruited to join this study. Patients with hepatosplenomegaly or isolated splenomegaly were analysed to exclude acid sphingomyelinase deficient Niemann-Pick disease and Gaucher disease by measuring acid sphingomyelinase and glucocerebrosidase enzyme activity of peripheral blood leukocytes before determination of plasma 7-KC levels. Infants with sustained cholestasis, with or without hepatosplenomegaly/hepatomegaly/splenomegaly were classified to group A; Individuals with hepatosplenomegaly/hepatomegaly/splenomegaly with or without a history of newborn jaundice were classified to group B; Individuals with hepatosplenomegaly/hepatomegaly/splenomegaly followed by psychomotor regression/retardation were also classified to group B; patients dominated with psychomotor regression/retardation, without palpable hepatosplenomegaly were classified to group C. There were 11 infants in group A, 124 patients in group B, and 167 patients in group C. As for the age, there were 45 individuals 15 years and older, 62 between 6 and 15 years old, 79 between 2 and 6 years old, 104 individuals between 3 months and 2 years old, and 12 under 3 months old.

### Plasma 7-KC levels

The plasma 7-KC level was evaluated as previously reported [21]. Blood was collected in EDTA or heparin tube with the caution to avoid hemolysis, transported within 4 hours at room temperature to our lab, and kept at 4°C. Plasma were then separated at the same day of sample collecting and frozen at -80°C before analysis. Internal standard solution of 100 μL (CND Isotopes, Quebec, Canada) was added to 25 μl plasma. The mixture was vigorously mixed and centrifuged at a speed of 13,300 rcf for 5 min at room temperature and then the supernatant was transferred to a 96-well plate. Analysis was performed on a UPLC system with a Xevo TQ MS detector (Waters, USA). 7-KC and d7-7-KC were detected using positive electrospray ionization through multiple reaction monitoring modes. The normal range of 7-KC is 0 ~ 12.3 ng/mL (95^th^ percentile equals to 12.3, Maximum is 22.8) [21].

### Clinical observations of patients with confirmed *NPC1* or *NPC2* gene mutation

Patient histories, physical examinations, and follow-ups were conducted by at least one of the listed authors. Medical history, including the initial onset of symptoms and the progression of the neurological symptoms until presentation at our outpatient clinic in Xinhua Hospital, was based on interviews with parents and patients’ past medical charts. All subjects were from nonconsanguineous families. Patients 6 and 7 were siblings, and the others were unrelated. We focused on the commonly occurring symptoms/conditions, such as jaundice, hepatosplenomegaly, vertical supranuclear gaze palsy, ataxia, gelastic cataplexy, seizure, gain/loss in psychomotor developmental milestones, and blood chemistry panels. For two patients (patients 4 and 11) liver enzyme tests performed in the perinatal period when jaundice was prominent are listed. For the rest of the patients, if multiple liver enzyme tests were performed during the disease course, the latest tests prior to presentation in our clinic were included.

### Sequencing of genomic DNA

An elevated level of 7-ketocholesterol was a prerequisite for molecular characterization of the *NPC1* or NPC2 genes, where the *NPC1* gene has priority over the *NPC2* gene. Genomic DNA was extracted from peripheral blood using the RelaxGene blood DNA isolation kit (DNA DP319-01, Tiangen Biotech Co. Ltd., Beijing, China) according to the manufacturer’s protocol. All exons and flanking regions of the *NPC1* gene were amplified using 23 primer pairs with exon 12–13, exon 15–16 amplified using one primer pair, respectively. The five exons of the *NPC2* gene were amplified by five primer pairs. All patients’ parental DNA samples were analysed to explore the mutation origin.

All primer sequences are available on request. All amplicons were bi-directionally sequenced, and the results were aligned with the *NPC1* gene reference sequence (NM_000271.4) and the *NPC2* gene reference sequence (XM_005267270.1). The novelty of all mutations were checked with the database (http://www.hgmd.org/) and the novel mutations were further checked in 100 normal Chinese to exclude rare benign variations.

## Results

### Determination of 7-KC levels

**Figure 1:**
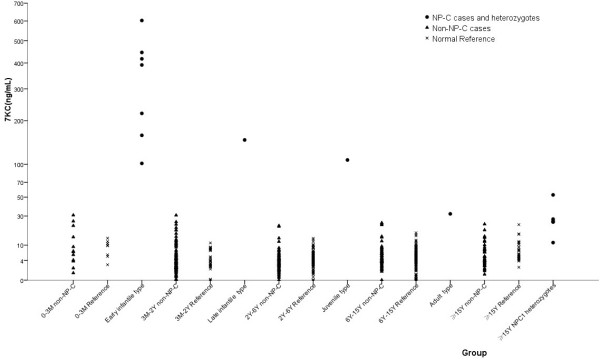
Plasma 7-KC levels in four subgroups of Chinese NP-C patients.

From 302 suspect patients we identified 12 individuals with marked elevated plasma 7-KC levels, which spanned from 32 to 602.7 ng/mL, with an average value 276.7 ng/mL (Table 1, Figure 1). These included 10 patients from group B, 2 patients from group C. These 12 patients were all confirmed to be NP-C by molecular genetic testing of the *NPC1* and *NPC2* genes.

**Table 1:** Clinical and molecular findings in 12 Chinese NP-C patients

**No.**	**Group**	**Gender**	**Initial manifestation/age**	**Major clinical signs**	**Age at neuropsychiatric onset**	**Age at diagnosis**	**Defected gene**	**Genotypes**	**Location**	**Predicted amino acid changes**	**ALT (IU/L)**	**AST (IU/L)**	**bone marrow biopsy**	**7-KC (ng/mL)**
1	**early infantile**	F	frequent falls, 1y	delayed motor/neuron development, splenomegaly	1 y	4 y	NPC1	c.1501G>T + c.1800delC	E9 + E12	p.D501Y + p.I601FfsX13	16	97	foam cells	391.6
2		F	splenomegaly, 6 m	splenomegaly, global developmental delay, diarrhea, seizure at 7 y, cerebral atrophy on brain imaging	1 y	8 y	NPC2	c.3G>C + IVS2 + 5G > A	E1 + IVS2	p.M1I + IVS2 + 5G > A	4	66	foam cells	220.8
3		M	splenomegaly, 1y	splenomegaly, frequent falls when started independent walking at 1.5 y, congenital cleft lip and palate, language delay, gelastic cataplexy from 2.5 y, positive family history	1.5 y	3 y	NPC1	c.416_417insC + c.1832A>G	E3 + E12	p.N140KfsX30 + p.D611G	10	72	foam cells	602.7
4		M	prolonged jaundice,15 ds after birth	prolonged jaundice, hepatosplenomegaly, frequent falls when started independent walking at 1 y, slower intelligence progression, psychomotor regression and gelastic cataplexy from 3 y	1 y	5 y 10 m	NPC1	c.2177G>C + c.3734_3735delCT	E14 + E24	p.R726T + p.P1245RfsX12	149*	301*	foam cells	445.8
5		M	delayed independent walk, 1y8m	slow motor development, hepatosplemegaly, psychomotor regression at 2 y	1 y 8 m	2 y 2 m	NPC1	c.2230_2231delGT + c.3734_3735delCT	E14 + E24	p.V744SfsX27 + p.P1245RfsX12	27	83	foam cells	417.5
6		F	frequent falls,1y3m	frequent falls when walking, gelastic cataplexy, motor regression at 3 y, splenomegaly	1 y 3 m	7y	NPC1	c.1553G>A + c.2795dupA	E9 + E18	p.R518Q + p.Y932X	15	75	foam cells	161.7
7		M	frequent falls, 1y8m	frequent falls when walking, gelastic cataplexy, splenomegaly, younger brother of patient 6	1 y 8 m	3y7m	NPC1	c.1553G>A + c.2795dupA	E9 + E18	p.R518Q + p.Y932X	N.D	N.D	N.D	101.6
8	**late infantile**	M	hepatosplenomegaly, 1y8m	hepatosplemegaly, thrombocytopenia, psychomotor regression at 2.5 y	2 y 6 m	1 y 8 m	NPC1	c.2302_2303insG + c.2912-3C>G	E15 + IVS19	p.V768GfsX4 + c.2912-3C > G	24	75	foam cells	150.6
9	**juvenile**	M	ataxia, 8ys	ataxia, progressively loss of memory, loss of language at 9 ys, seizure from 12 y, thrombocytopenia without splenomegaly	8 y	13 y	NPC1	c. 1502A>T + c. 3634G>T	E9 + E24	p.D501V + p.V1212L	10	59	N.D	108
10	**adult**	M	visual and auditory hallucination, 16 ys	schizophrenia-like psychosis, splenomegaly deteced by abdominal ultrasound, intestinal constipation	16 y	20 y	NPC1	c.2366G>A + c.2972_2973delAG	E15 + E20	p.R789H + p.Q991RfsX15	34.7	39.6	N.D	32
11	**unknown**	M	delayed jaundice regression, 1 m	delayed jaundice regression, increasing splenomegaly, mild hepatomegaly	N.A	14 m	NPC1	c.2054T>C + c.2128C>T	E13 + E13	p.I685T + p.Q710X	59*	145*	foam cells	456.3
12		F	splenomegaly, 3y3m	isolated splenomegaly	N.A	3y5m	NPC1	c.1030del>T + c.2861C>T	E8 + E19	p.S344KfsX105 + p.S954L	N.D	N.D	N.D	232.3

* indicated measurement in newborn with jaudice. N.A.indicated not applicable. N.D.indicated undone.

**Figure 2:**
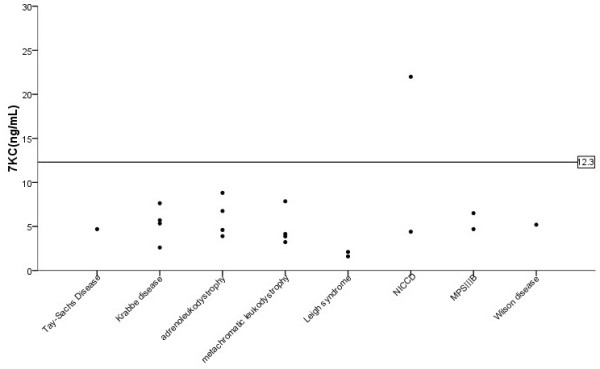
Plasma 7-KC levels in patients with confirmed alternative diagnosis in the non-NP-C patients.

The 7-KC in the non-NP-C cases were 5.5 ± 5.2 ng/ml (mean ± SD, n = 290). The highest 7-KC value in the non-NP-C group is 30.8 ng/ml, very close to the lowest 7-KC value (32 ng/ml, patient 10) in the NP-C patients. This was an infant with cholestatic jaundice without confirmed diagnosis, who was later excluded as NP-C or a heterozygote of *NPC1/NPC2* gene by molecular analysis. The 7-KC values in the confirmed heterozygotes, parents of NPC1 patients, were 20.9 ± 17.2 ng/ml (mean ± SD, n = 6). Since most non-NP-C patients were not continuously followed in our clinics, we only knew a few of them with clear diagnosis in the non-NP-C cases. There was one patient with Tay-Sachs disease, four patients with Krabbe disease, four patients with adrenoleukodystrophy, four patients with metachromatic leukodystrophy, two patients with Leigh syndrome, two patients with Neonatal intrahepatic cholestasis caused by citrin deficiency (NICCD), two patients with mucopolysacharidosis type IIIB and one patient with Wilson disease (Figure 2). The 7-KC value in one patient with NICCD was 22 ng/ml, which was above the border line and lower than the maximum value in normal reference.

The confirmed NP-C patients were then categorized into four subtypes based on presence of neurological symptoms and the age at neurological onset. There were seven patients in the early-infantile subtype. For the late-infantile, juvenile, and adult subtypes, each has one patient respectively. No patient in the perinatal subtype was identified. Consistent with previous findings, there was a tendency that 7-KC levels inversely correlated with the age of disease onset although there were limited numbers of patients in each group (Figure 1 and Table 1) [20].

We identified more than half (7/12) of the patients in our cohort as fulfilling the characteristics of early infantile subtype, which was different from previous reports of Chinese NP-C patients, where all patients were categorized into late infantile and juvenile subtypes [17,18,23]. This is also the first time that an adult subtype of NP-C has been reported in Chinese patients.

Two individuals without neurological manifestations were confirmed to be NP-C (numbers 11 and 12). Vertical supranuclear gaze palsy was absent in these two patients after repeated careful observation.

### Clinical findings of confirmed NP-C patients

In line with previous publications, 10 of 12 patients had visceral presentations, isolated splenomegaly or hepatosplenomegaly. For patient 10, who belonged to the adult subtype, the spleen was not palpated under the left ribs, while spleen thickness was increased per ultrasound. For patient 9, who was a juvenile form of NP-C, the abdominal ultrasound did not detect splenomegaly. Only two children (patients 4 and 11, 16.7%) had cholestatic liver disease together with hepatosplenomegaly, which was strikingly lower than a previous report, where 39% ~ 65% of NP-C patients had this symptom in infancy [10,16,24]. The neurological symptoms, if present, were always characteristics of ataxia, psychomotor regression, gelastic cataplexy, seizure, and psychosis. Bone marrow biopsy has a high diagnostic value. Foam cells were identified in all patients who undertook this procedure.

We found that AST was usually mildly elevated (higher than 40 IU/L, but lower than 100 IU/L), and ALT was in a normal range (<40 IU/L), except for the two measurements taken when jaundice presented in patients 4 and 11. In all of these 10 patients with liver enzyme test results available, AST had a significantly higher value than ALT (average value, 101.3 IU/L verse 32.6 IU/L, p < 0.001).

### Genetic examinations

As for the molecular diagnosis of the 12 Chinese NP-C patients, 11 patients had a defective *NPC1* gene, whereas only one (patient 2) had a defective *NPC2* gene. This observation is consistent with data from other ethnic groups that the majority of patients have a mutated *NPC1* gene [13,25]. This is also the first time that a new set of *NPC2* gene mutations has been identified in a Chinese patient. In this patient, a reported mutation IVS2 + 5G > A [26] and a novel mutation c. 3G > C (p.M1I) have been identified.

**Figure 3:**
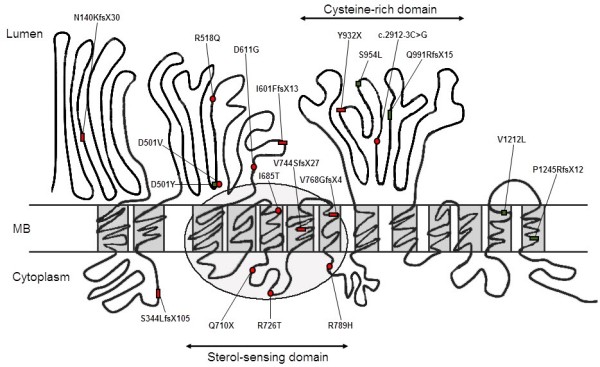
**Localization of mutation identified in 12 Chinese NP-C patients draw on the schematic NPC1 protein model**[29]**.** Red circle: novel exonic point mutations; Red rectangle: novel small insertion/deletions; Green square: reported exonic point mutations; Green rectangle: reported small insertion/deletions.

Both mutant alleles were found on the *NPC1* gene from 11 patients (Table 1). Surprisingly, 10 (10/22, 45%) were small deletions/insertions, which were presumed to cause frame shifts and premature protein truncations (Figure 3). Except for a novel intronic mutation c.2912-3C > G, predicted to alter the splicing acceptor site of intron 19 by ASSP (http://wangcomputing.com/assp/index.html) and MutationTaster (http://www.mutationtaster.org/), the rest of the 11 mutations (11/22, 50%) were exonic point changes resulting in codon replacement. Nearly all of the mutations were distributed in axons post 7, except one in exon 3 (Table 1).

The majority of small deletions/insertions were reported here for the first time, including c.1800delC, c.416_417insC, c.2302_2303insG, c.2230_2231delGT, c.1030delT, and c.2795dupA. Two small deletions, c.3734_3735delCT and c.2972_2973delAG, had been published previously [27,28]. The c.3734_3735delCT was the only recurring mutant in this group, presenting twice (patients 4 and 5), indicating that this may be a comparatively common mutation in the Chinese population.

Among exonic point changes, three mutants were previously published, c. 1501G>T (p.D501Y) [17], c.3634G>T (p.V1212L) [18], and c.2861C>T (p.S954L) [29]. We did not find the hot mutations including p.I1061T, p.P1007A, and p.G992W that are the most recurrent in the Western population [13]. In addition to one patient in this paper, four unrelated Chinese patients with p.V1212L in heterozygous state have been described [11,18], indicating that this may be another comparatively common mutation in the Chinese population.

The seven novel exonic point mutations were c. 1502A>T (p.D501V), c.1553G>A (p.R518Q), c.A1832A>G (p.D611G), c.2054T>C (p.I685T), c.2128C>T(p.Q710X), c.2177G>C (p.R726T), c.2366G>A (p.R789H). Except for the stop codon mutation p.Q710X, the rest were exonic missense mutations, which were highly likely to be pathogenic, as predicted by MutationTaster. All novel mutations have been submitted to the public database ClinVar.

## Discussion

The current diagnosis of NP-C was mainly filipin staining for unesterified cholesterol of cultured fibroblasts obtained through invasive skin biopsy. The positive individuals were then followed by genetic testing. However, filipin assay is time-consuming and cannot discriminate some cases with a “variant” filipin pattern from normal controls [5]. The filipin assay is performed in only a few centres world-wide. Some small diagnostic centres perform mutation analysis alone, where two mutant alleles are necessary to confirm the diagnosis. But in about 10-15% cases, routine DNA sequence failed to identify mutations on both alleles, especially when there were large deletions involving *NPC1*[30]. Furthermore, mutation analysis alone could not differentiate disease-causing alleles from polymorphisms when confronting unreported nucleotide changes.

When 7-KC and 3β,5α,6β-triol were found markedly increased in the plasma of human NPC1 subjects and NPC1 mouse model, and were decreased under therapy, they were regarded as NPC1 disease-specific biochemical markers and will replace the filipin assay as the diagnostic standard for NPC1 disease. Since 3β,5α,6β-triol was not readily detectable by a non-derivatized method, we concentrated on 7-KC detection [21]. We found that 7-KC was in normal range in patients with MPS II, GSD II, Gaucher disease, Metachromatic leukodystrophy, and Krabbe disease, and was markedly elevated in patients with ASM deficient NPD and NPC disease [21]. Here we employed this test in populations with hepatosplenomegaly/isolated splenomegaly, excluding ASM deficient NPD, and in populations with neonatal cholestasis, phychomotor regression/retardation, where NP-C disease may have a comparatively high prevalence.

Since 7-KC assay could be performed in a high throughput manner in our lab, we did not calculate score of the suspicion index [31] when recruiting subjects to this study to avoid missing atypical patients. We identified 12 individuals with high levels of plasma 7-KC, who were then confirmed to be NP-C by genetic testing, indicating a strong specific correlation between elevated 7-KC and NP-C. Our data also demonstrate that one NP-C patient due to *NPC2* gene mutation also had high 7-KC levels, which was reasonable considering the concert signaling pathway of NPC1 and NPC2. Since the 7-KC value in the adult type could be as low as those in the NPC1 carriers, in the highly suspect adult patients, even if the detected 7-KC value is at the border line, it is still necessary to follow up with the *NPC1* and *NPC2* gene analysis to avoid any potential misdiagnosis.

The 7-KC positive rate is about 4% in our tested population. It seems lower than reported prevalence of NPC1 disease that approaches 8% in neonates with jaundice in one medical centre [6]. The comparatively low positive rate may reflect the loose criteria in subject recruiting or the true lower prevalence of NP-C in Chinese than that in Western countries since there were no hot mutations in reported Chinese cases, including cases in this report.

As expected, with this diagnostic flowchart, we identified two patients (numbers 11 and 12) without obvious neurological impairment. Since current data are suggestive of a correlation of high 7-KC with disease severity, these two patients were predicted to have an early or late infantile type with 7-KC 456.3 ng/ml and 232.3 ng/mL, respectively. Further clinical following-up will be needed to confirm this speculation. But if the therapeutic drug were available to these two patients at this stage of the disease, their prognosis might be much better than its natural course [12].

Most publications emphasized the neurodegeneration characteristics of NP-C disease, apart from the prolonged jaundice in the neonatal period. In a study of paediatric NP-C disease with particular reference to liver disease, half of the NP-C children who survived the neonatal jaundice had persistent liver disease with elevated aminotransferase values while the other half had normal aminotransferase activity. In NP-C cat and mouse models, elevated ALT and AST were also consistently observed [32,33]. These data support liver involvement as an important feature of NP-C disease phenotype in addition to its distinctive neurological degeneration.

We also paid special attention to routine biochemical panels. We observed that both ALT and AST were elevated in NP-C patients with jaundice (patient 4 and 11), while only mildly elevated AST was observed in NP-C patients without jaundice. In both situations, the AST was always higher than ALT. Elevated AST was consistently observed in a patient without hepatosplenomegaly (patient 9). It is well known that ALT is a cytosolic enzyme specific for increases in hepatocellular permeability, and AST is located in both the cytosol and mitochondria, which would increase in serum with hepatocellular or muscle damage. Mildly elevated AST with normal ALT may mean more severe damage of unesterified cholesterol or its byproduct oxysterols to hepatocyte mitochondria than to hepatocyte membrane. Cellular oxidative stress to mitochondria had been demonstrated in NPC1-deficient human cells [34] and NP-C mouse models [35], which provided a basis for our hypothesis.

Our data showed that small deletions/insertions constituted nearly half of all mutant alleles, which different from results obtained in previous molecular studies of Chinese NP-C patients and other ethnic groups, where point mutations leading to amino acid missense changes constituted the majority of all mutations [15,17,18,25]. Because frame shift mutations correlated with the most severe neurological presentation, it is consistent with the patients’ clinical courses that six out of 11 NPC1 mutated patients (patients 1, 3, 4, 5, 6, and 7) belonged to the early infantile form. Our data may be biased towards very young children, since two third individuals in the whole cohort were under 6 years old.

Four point mutations on the sterol-sensing domain, p.I685T, p.Q710X, p.R726T, and p.R789H, were detected. The combination of a small deletion c.3734_3735delCT and p.A726T in an early infantile form of NP-C disease (patient 4) corroborated that missense mutations located in the sterol-sensing domain are very deleterious [29]. While two severe mutations, c.2972_2973delAG and p.R789H, were detected in an adult form (patient 10). These observations showed the complexity of phenotype and genotype correlation in the NP-C disease. Two mutant alleles of patients 11, p.I685T and p.Q710X, were on the sterol-sensing domain, indicating that he may experience a severe form of NP-C. The high 7-KC level (456.3 ng/ml) of this patient provided additional evidence of a severe clinical course.

## Summary

With the help of the plasma biomarker 7-KC, we had made rapid diagnoses of NP-C in a cohort of Chinese patients. If plasmatic 7-KC assay becomes widely available in the future, it might be used as a first screening for all types of Niemann-Pick disease. In addition to the characteristic somatic and neurologic presentations and foamy cells in bone marrow biopsy, the mildly elevated AST levels compared with normal ALT levels may be easy recognizable serum changes in NP-C disease without cholestasis before screening of 7-KC. A total of 14 novel mutations in the *NPC1* gene and one novel mutation in the *NPC2* gene have been identified. With increasing awareness of this devastating disease, and using the rapid and reliable plasma biomarker 7-KC, we envision that more patients should receive a timely diagnosis and treatment in the future.

## Competing interests

The authors declared that they have no competing interests.

## Authors’ contributions

ZH and GX conceived of the study. Na Lin determined the plasma 7-KC levels of patients. WY and YR carried out the molecular genetic studies. ZH, YJ, QW, HL, GX took care of the patients. ZH participated in the sequence alignment and drafted the manuscript. All authors read and approved the final manuscript.
